# Prokineticin 2 *via* Calcium-Sensing Receptor Activated NLRP3 Inflammasome Pathway in the Testicular Macrophages of Uropathogenic *Escherichia coli-*Induced Orchitis

**DOI:** 10.3389/fimmu.2020.570872

**Published:** 2020-10-23

**Authors:** Yufang Su, Yuan Zhang, Zhiyong Hu, Liting He, Wei Wang, Jia Xu, Zunpan Fan, Chunyan Liu, Huiping Zhang, Kai Zhao

**Affiliations:** Institute of Reproductive Health, Tongji Medical College, Huazhong University of Science and Technology, Wuhan, China

**Keywords:** calcium-sensing receptor, testicular macrophages, inflammasome, orchitis, prokineticin 2

## Abstract

Reproductive tract infections contribute to the development of testicular inflammatory lesions, leading to male infertility. Previous research shows that the activation of the NLRP3 inflammasome in orchitis promotes the secretion and maturation of IL-1β and, thus, decreases male fertility. The calcium-sensing receptor (CaSR) is closely related to the secretion of proinflammatory cytokines. An increase in the CaSR level promotes the assembly and activation of the NLRP3 inflammasome. However, the role of CaSRs in orchitis is unknown. We first constructed a uropathogenic *Escherichia Coli* (UPEC) rat orchitis model and then detected the expression of CaSR and NLRP3 inflammatory pathway proteins in testicular macrophages (TM) through RT-PCR and WB, calcium levels in TM through flow cytometry, and proinflammatory factor IL-1β through ELISA. In addition, testosterone levels in the serum samples were detected using liquid chromatography–mass spectrometry (LC-MS). Here, we show that CaSR upregulation after infection in TM in a rat model of UPEC induces the activation of the NLRP3 inflammasome pathway and thereby enhances IL-1β secretion and reduces the testosterone level in the blood. Moreover, CaSR inhibitors can alleviate inflammatory impairment. After UPEC challenge *in vitro*, CaSR promoted NLRP3 expression and released IL-1β cleaved from TM into the supernatant. Overall, elevated CaSR levels in TM in testes with UPEC-induced orchitis may impair testosterone synthesis through the activation of the NLRP3 pathway and PK2 is an upstream regulatory protein of CaSR. Our research further shows the underlying mechanisms of inflammation-related male infertility and provides anti-inflammatory therapeutic targets for male infertility.

## Introduction

Decline in male sperm quality and male infertility is a worldwide concern. Approximately 15% of male infertility cases are related to the inflammation of the reproductive system (1), and approximately 60% of such cases are caused by uropathogenic *Escherichia* *Coli* (UPEC) (2). Testicular impairment is usually caused by the immune system when it is killing pathogenic bacteria during an infection rather than by the direct toxicities of pathogens and their secretions to testicular cells (3). Therefore, studying the immune regulation of inflammation-related male infertility is of great significance to clinical diagnosis and treatment.

Testicular macrophages (TM) comprise the largest proportion of immune cells in the interstitial space of the testis (4). Rat TM account for approximately 20% of total immune cells, constituting the first line of defense against pathogens. Macrophages and NLRP3 inflammasomes are involved in the onset and development of orchitis (5). NLRP3 inflammasomes are composed of cytoplasmic sensor molecules, such as the PYD domain-containing protein 3 (NLRP3), adaptor proteins (e.g., caspase-recruiting domain [ASC] of apoptosis-associated speck-like proteins), and effector proteins (e.g., pre-caspase-1) (6–8). NLRP3 and ASC promote the cleavage of pro-caspase-1 and form an active complex, which triggers the cleavage of pro-IL-1β into mature IL-1β (9). In a murine model of UPEC orchitis, NLRP3, ASC, caspase-1, and IL-1β are clearly elevated in TM (10–12).

The calcium-sensing receptor (CaSR) can sense small changes in Ca^2+^ concentration, mediate signal transduction in the cytoplasm, promote the recruitment and assembly of NLRP3 inflammasomes, and cause an inflammatory response (13). Increasing evidence shows that the elevated circulating levels of proinflammatory cytokines are accompanied by changes in Ca^2+^ homeostasis (14). CaSR is expressed in BMDNs, promotes the assembly of NLRP3 inflammasomes by regulating Ca^2+^ concentration, and modulates the secretion and maturation of IL-1β (15). CaSR activates NLRP3 inflammasomes through the ERK1/2 signaling pathway in the preadipocyte line LS14 (16). However, the role of CaSR in UPEC-induced orchitis macrophages remains unclear.

Given the potential relationship between CaSR and NLRP3, we concluded that CaSR is closely related to UPEC-induced testicular inflammation. Based on our previous findings that PK2 promotes IL-1β secretion through the NLRP3 pathway (12), we speculated that PK2 and CaSR have a close relationship. In this study, we focused on the effect of CaSR on IL-1β secretion in TM during a UPEC infection to reveal the molecular mechanism that may ultimately damage male fertility.

## Materials and Methods

### Animals

Adult male Wistar rats (8–10 weeks) were purchased from the Animal Center of Tongji Medical College. The rats were raised at 22°C in a 12 h light/12 h dark cycle and fed with standard food pellets and water. The study was conducted in strict accordance with the guidelines approved by the Animal Care and Use Committee of Tongji Medical College, Huazhong University of Science and Technology.

### Bacterial Reproduction and Detection

The UPEC strain CFT073 (NCBI: AE014075, NC_004431) bacterial culture was shaken in an LB liquid medium and then grown to the exponential phase (OD600 = 0.6–0.8). The UPEC culture was centrifuged at 4000x*g* for 10 min at room temperature. The obtained pellets were washed with PBS and stored in DMEM 0F12:1. Then, a 9-cm LB solid medium plate was prepared, and the UPEC bacterial solution was diluted at different concentration gradients. Approximately 10 µL from each gradient solution was smeared evenly on the plate. Finally, the total number of CFU of UPEC was calculated.

Detection of bacteria in the testes of UPEC model rats: A small portion of each testis tissue sample was ground into a homogenate. After dilution with physiological saline, 10 µL of the homogenate was evenly spread on an LB solid medium, and the formation of UPEC CFU was observed on the next day.

UPEC-treated cells *in vitro*: Cells were infected with UPEC (Moi = 20) for 2 h.

### UPEC Rat Model

After the rats were anesthetized, the testes and epididymides were fully exposed for the location of the vasa deferentia. Approximately 50 µL of 4 × 10^6^ CFU UPEC CFT073 bacterial solution diluted with saline was injected into the vas deferens near the tail of the epididymis on both sides. The control group was injected with 50 µL of saline. After constructing the UPEC rat orchitis model, NPS2143 containing 0.5% DMSO was injected *in situ* into the testis of the rat the next day, and the concentration was 10 mg/kg according to the body weight of each rat.

### Sample Collection

Collection of testis and epididymis tissues: Laboratory equipment was cleaned and disinfected at 125°C. After the rats were killed, the testes and epididymides were exposed immediately, and any damage to the seminiferous tubules was prevented by carefully removing the tissues.

For the collection of the testicular interstitial fluid, a 2-mm incision was made at the end of each testis, and then the white membrane was sutured at the top of the testis with four surgical threads. The testis was suspended in four refrigerators in a 15-mL centrifuge tube for 16 h and centrifuged at a speed of 200 RPM for 3 min. The resulting transparent tissue fluid was collected and stored in a refrigerator at −80°C.

Collection of the supernatant of the primary macrophages: Primary macrophages from each group adhered to the wall for 30–40 min. Each culture medium was changed for the stimulation of adherent macrophages and subsequent collection of supernatants.

### Flow Cytometry

A stain buffer (100 µL) containing 1% BSA was used in the preparation of single-cell suspensions from primary TM. TM were broken, placed in an EP tube, and centrifuged at 300x*g* at 4°C for 10 min. The resulting pellets were collected, and impurities were removed by resuspending the primary macrophages. Mouse anti-CD68-Alexa Fluor 488 (Bio-Rad, USA) and mouse anti-CD45-PE/Cy7 (BioLegend, USA) fluorescent antibody-labeled macrophages were prepared and incubated at 4°C in the dark for 50 min. The primary TM were washed again and centrifuged at 300x*g* for 10 min. The resulting pellets were collected, and the primary TM were resuspended after incubation for on-board testing.

Calcium ion detection: 350 µL of interstitial single-cell suspension (approximately 1×10^6^ cells) and 2 μL of mouse anti-CD68-Alexa Fluor 488 (Bio-Rad, USA) and fluo4-am were used. After the exclusion of cell aggregation and bimodality according to the side scatter A (SSC-A) and SSC-H plots, flow cytometry analysis was performed using a flow cytometer (BD LSR II, USA), and data were analyzed using FlowJo version X.

### Testicular Histopathology

Fresh testes from each group were removed and immediately placed in Bouins solution for 48 h. Testicular tissues were embedded in paraffin. The wax blocks were completely solidified and then cut into thin slices on a machine. The incised testicular tissues were dewaxed, stained with hematoxylin and eosin, and observed under a microscope.

Testes the same size as mung beans were collected form each group within 2 min and placed in a fixative solution. The testis microstructure was observed under an electron microscope.

### Sperm Count and Sperm Forward-Movement Detection

The rats were anesthetized and sacrificed. Epididymal samples were collected, cut into small pieces, incubated in an F10 medium for 20 min, and counted under the microscope.

### Cell Isolation

Isolation of adult Wistar rat TM. The testes were decapsulated and digested with 1 mg/mL collagenase I (Sigma, USA) at 34°C for 20 min. Then, mesenchymal cells and seminiferous tubules were separated through 200-mesh filtration. The cells were cultured in DMEM F12:1 (Life Technologies, USA). After 40 min, nonadherent cells were removed by culture medium washing. The remaining adherent interstitial cells were primarily TM.

### Cell Viability Measurements

After the UPEC bacteria with MOI=20 stimulated the J774A.1 and Raw264.7 macrophage cell lines for 2 h, the cells were treated with the NPS2143 inhibitor for 4 h. According to the manufacturer’s instructions, cell viability was assessed by Pierce LDH cytotoxicity assay (Beyotime, China) and neutral red toxicity assay (Beyotime, China) (17).

### RT-PCR

Total RNA was isolated from TM using TRIzol reagent (Invitrogen, USA). Reverse transcription was performed using a PrimeScript™ RT kit. PCR was performed in 20 μL of a reaction mixture containing 2 μL of cDNA, 0.8 μL of forward primer, 0.8 μL of reverse primer (**Table 1**), and 10 μLSYBR Green PCR MasterMix. The amplification conditions were initial denaturation at 95°C for 30 s, 40 cycles of denaturation at 95°C for 5 s, annealing at 60°C for 30 s, and elongation at 72°C for 30 s. The 2^-ΔΔCt^ method was used in normalizing the relative expression level of the target gene to the relative expression level of the control.

**Table 1:** Sequences of primer pairs used in the real-time quantitative PCR reactions.

Gene	Primer sequences (from 5’ to 3’)	Size (bp)
β-actin	F: GAGAGGGAAATCGTGCGT	93
	R: GGAGGAAGAGGATGCGG	
CaSR	F: CTCCATTCCCTCCTCCTCCATCAG	82
	R: TTGCTGTTGCTTCTGCCTCTCTG	
PK2	F: CAAGGACTCTCAGTGTGGA	128
	R: AAAATGGAACTTTCCGAGTC	

### Western Blot Analysis

Tissue and cell proteins were lysed on ice with radioimmunoprecipitation assay lysis buffer (Cwbio, Taizhou, China). Total proteins were collected after centrifugation. Then, 5 µL of 5× loading buffer was added to the proteins, which were denatured at 98°C and stored in a refrigerator at −20°C. Colloidal preparation, electrophoresis, membrane transfer, sealing, and other operations were carried out successively according to the kit’s instructions. The following primary antibodies were used during incubation: goat anti-IL-1β polyclonal antibody (1:1000, R & D Systems, USA), rabbit anti-CaSR polyclonal antibody (6D4, 1:500, Santa Cruz Biotechnology, USA), rabbit anti-NLRP3 polyclonal antibody (1:500, Novus, USA), mouse anti-caspase-1 monoclonal antibody (1:500, Novus, USA), and mouse anti-β-actin polyclonal antibody (1:500, Boster, China). Protein bands were detected using ECL (Pierce, USA).

### Enzyme-Linked Immunosorbent Assay

According to the manufacturer’s instructions, the levels of IL-1β in the sera and supernatants were assessed using ELISA kits from R & D Systems (USA).

### Immunofluorescence

Cells seeded in coverslips were washed with PBS and then fixed with prechilled 4% formaldehyde. Subsequently, the cells were blocked with 5% normal goat serum for 1 h at room temperature and then treated with rabbit anti-CaSR polyclonal antibody (1:200, Abcam, USA), PK2 polyclonal antibody (1:200, Abcam, USA), and rabbit anti-CaSR polyclonal antibody (1:500, Abcam, USA) and incubated overnight. The cells were washed twice with PBS and then treated with the following secondary antibodies: donkey antimouse IgG H & L (Alexa Fluoro^®^ 647, 1:500, Abcam, USA) and goat antirabbit IgG H & L (FITC, 1:500, Abcam, USA) and left to stand at room temperature for 1 h. Nuclei were stained with DAPI.

### Testosterone Test

For liquid chromatography–mass spectrometry (LC-MS), 200 μL of each sample was mixed with 800 μL of precooled methanol/acetonitrile (1:1) for the precipitation of the proteins. The mixtures were centrifuged at 15,000x*g* for 4 min at 4°C. Then, the supernatants were collected and dried under vacuum. A solution containing acetonitrile and water (1:1; 100 μL) was added. The resulting solution was centrifuged at 14,000x*g* for 15 min at 4°C. The treated supernatants were analyzed using a liquid chromatograph LC-30A (SHIMADZU, Japan) and 4500 QQQ mass spectrometer (AB Sciex, USA).

### pCMV-HA-PK2 Expression Plasmid

A pCMV-HA-PK2 expression plasmid was constructed (18). The recombinant plasmid was transiently transfected into J774A.1 and RAW264.7 macrophage cell lines and TM-3 cells with Lipofectamine 3000 (Invitrogen). Briefly, transfection complexes (including the optimized concentration of the plasmid and Lipofectamine 3000) were transferred to 80% fused TM-3 cells for 6 h, and then the cells were washed and further cultured in DMEM for 42 h.

### Statistical Analysis

Statistical analysis was performed using the Social Science Statistics Package (SPSS) 18.0 and GraphPad Prism 5.0. Data were expressed as means and standard errors of the mean (SEM). Differences between two groups were analyzed through unpaired *t*-test. One-way analysis of variance and Tukey’s HSD *post hoc* test were used in measuring differences between groups. *P* < 0.05 were considered significant.

## Results

### Clear Decrease in Sperm Quality in UPEC Model Rats

**Figure 1:**
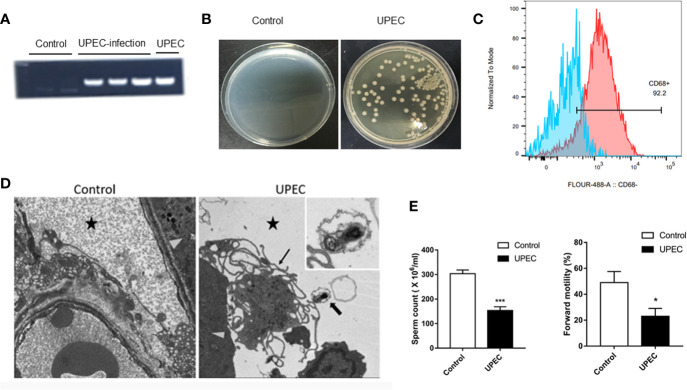
The UPEC orchitis model was successfully constructed. **(A)** The expression of the PaPC gene in the control group, UPEC infection group, and the positive control group (*n*=5/group). **(B)** The growth of the colonies after homogenization and plating of testis tissues in the control and infection groups (*n*=5/group). **(C)** CD68 flow antibody was used in labeling primary TM (*n*=3/group). **(D)** The location of UPEC in the testes in the infected group. The triangular icon represents the seminiferous tubule area, the five-pointed star icon represents the interstitial area, the thick arrow indicates UPEC, and the thin arrow indicates the primary TM. The picture on the left is the infected group at ×1700 magnification, and the right is the infected group at ×5000 magnification (*n*=5/group). **(E)** Comparison of sperm count and forward sperm motility in normal saline control and UPEC infection groups (*n*=3/group). *P < 0.05, ***P < 0.001 (*t*-test).

We established a rat UPEC model according to previous methods. To verify the success of model construction, we compared the model using the control group. We found that the UPEC content in the seminal plasma of the injection group increased markedly (**Figure 1A**). Moreover, the testes were mashed and smeared on a bacterial plate. The generation of strains was visible to the naked eye (**Figure 1B**). Flow analysis showed that the purity of the primary macrophages extracted was as high as 92.2% (**Figure 1C**). To verify that UPEC bacteria can enter the testicular interstitium, we observed the complete form of UPEC bacteria under an electron microscope (**Figure 1D**). Semen parameter analysis showed that the total sperm count and forward motility of the sperm in the UPEC group decreased (**Figure 1E**). All these results indicate that the UPEC model was successfully constructed, and the sperm motility of the rats decreased visibly after infection.

### CaSR Upregulates TM in Rat Testes With UPEC-Induced Orchitis

**Figure 2:**
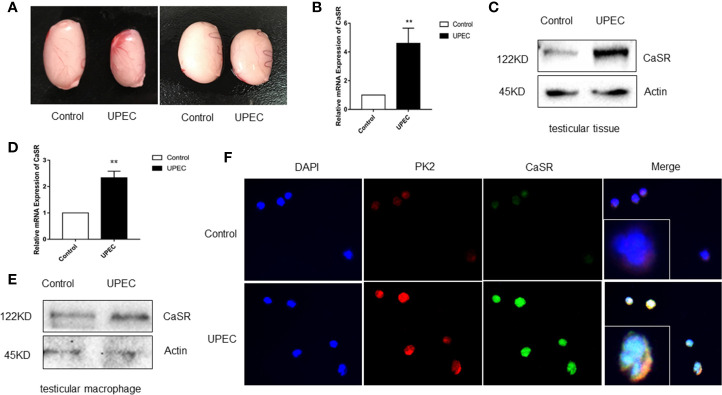
CaSR expression was increased in UPEC-infected rat TM. **(A)** Comparison of testicle size between the saline control and UPEC infection groups (*n*=5/group). **(B)** CaSR mRNA expression in the testis tissues of the normal saline control and UPEC infected groups (*P* < 0.01) (*n*=5/group). **(C)** CaSR protein content in the testis tissues of the two groups of rats. **(D)** The expression of CaSR mRNA in the testes of two groups in primary macrophages (*P* < 0.01) (*n*=5/group). **(E)** CaSR protein expression in the primary testicular macrophages of the two groups of rats. **(F)** Localization of CaSR in primary testicular macrophages. **P < 0.01 (*t*-test).

The role of CaSR in UPEC-induced orchitis was investigated. Seven days after UPEC infection, testicular volume decreased in the UPEC group (**Figure 2A**). The level of the *CaSR* gene was clearly elevated in the testicular group of the UPEC-infected rats (**Figure 2B**), and the expression level of the CaSR protein visibly increased (**Figure 2C**). Given that TM is the most abundant immune cell in the testicular interstitium, UPEC invasion and localization were detected. We focused on TM and isolated TM from UPEC-infected rats. The results show that CaSR gene and protein levels increased in the TM cells of the UPEC group (**Figures 2D, E**). Our previous results show that PK2 was upregulated in the TM of UPEC-induced rats. Our previous results indicate that PK2 is expressed in the rat TM nucleus, and UPEC infection induces the secretion of PK2 in the nucleus into the cytoplasm to play a proinflammatory role. Therefore, we identified CaSR in TM by positioning PK2 in rat TM localization and immunofluorescence results show that CaSR was expressed in the TM nuclei of the control rats, and CaSR fluorescence increased after UPEC infection. However, UPEC infection induced CaSR expression in the cytoplasm. This result suggests that CaSR protein is involved in efficient production (**Figure 2F**). In conclusion, CaSR is highly expressed in the TM of rats infected with UPEC.

### CaSR Inhibitor NPS2143 Relieves Orchitis Caused by UPEC

**Figure 3:**
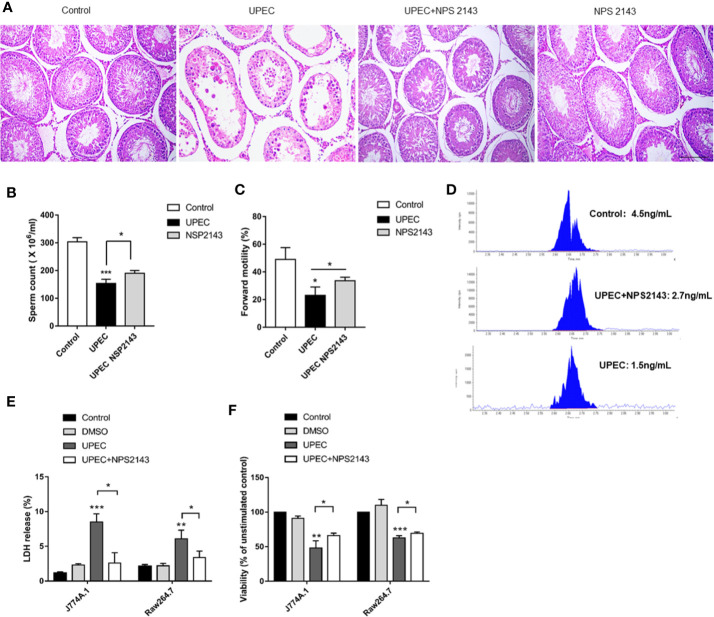
CaSR inhibitor NPS-2143 can reduce UPEC-induced testicular inflammatory damage. **(A)** The morphology of the testicular tissues in the normal saline control group was intact; UPEC infection group showed obvious the irregular arrangement of spermatogenic epithelium, infiltration of inflammatory cells in the interstitium, and the destruction of many spermatogenic cell structures; UPEC+NPS-2143 inhibitor group. The inflammatory injury of the testis was relieved; the NPS-2143 negative control group showed no significant changes compared with the saline control group. Scale bar 100 um (*n*=5/group). **(B)** Comparison of the total number of sperm in normal saline control group, UPEC infection group, and NPS2143 rats (*n*=5/group). **(C)** The comparison of the forward movement speed of spermatozoa in the three groups (*n*=5/group). **(D)** The testosterone levels in the sera of rats were detected by LC-MS. **(E)** Assessment of LDH release from macrophage cell lines J774A.1 and Raw264.7 by UPEC strain. **(F)** Neutral red stains the macrophage cell lines J774A.1 and Raw264.7 in each group. *P < 0.05, **P < 0.01, ***P < 0.001 (*t*-test).

Given the proinflammatory effects of CaSR, its effects on inflammatory response and the male fertility process were studied. The CaSR inhibitor NPS2143 was injected into the testes of the UPEC-infected rats. CaSR activity was inhibited after injection. After UPEC infection, the renal tubules were inflamed, and testicular spermatogenic cells showed abnormal morphology. However, after treatment with NPS2143, inflammation was alleviated, and the severity of the damage to germ cells decreased. In the rats treated with NPS2143 alone, no significant changes in testicular morphology and parameters related to male reproduction were observed (**Figure 3A**). In addition, UPEC had a negative effect on sperm count and forward motility, and the UPEC + NPS2143 group showed the partial recovery of these factors (**Figures 3B, C**). The LC-MS results show that UPEC reduced the production of testosterone in the serum samples, and the UPEC + NPS2143 group displayed a partial recovery (**Figure 3D**). The UPEC strain was used to stimulate the macrophage cell lines J774A.1 and Raw264.7 to assess cell viability. The results show that, compared with the control group, the LDH released by the macrophage cell line increased in the UPEC group (**Figure 3E**), and the neutral red vitality test showed a significant decrease in cell metabolic activity (**Figure 3F**). However, NPS2143 inhibitors can be used to a certain extent to restore cell viability (**Figures 3E, F**). Our findings indicate that CaSR promoted testicular inflammation, thereby impairing male reproductive capacity, but the CaSR antagonist NPS2143 reduced inflammation and promoted sperm count recovery, forward motility, and testosterone production.

### CaSR Regulates Ca^2+^ and Activates the NLRP3 Pathway

**Figure 4:**
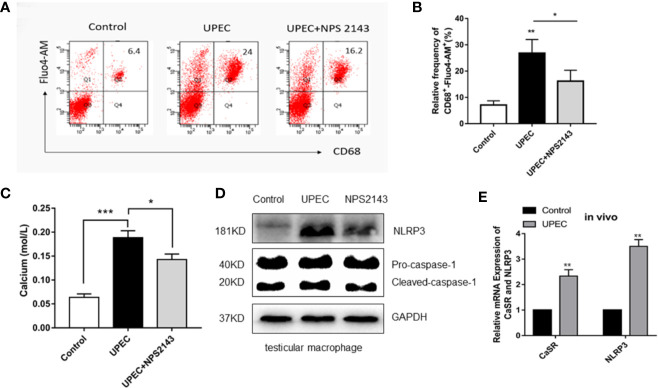
CaSR promotes the recruitment and assembly of NLRP3 inflammasomes through Ca^2+^. **(A)** The level of calcium ion in primary TM of each group was detected by flow cytometry (*n*=3/group). **(B)** Statistical analysis was performed on the results of the convection detection of calcium ion levels in the primary TM of each group. **(C)** The calcium level of each group labeled with the fluorescent indicator Fluo-am was detected using a microplate reader (*n*=3/group). **(D)** TM were isolated from the testes of rats in the control group and 7 days after UPEC treatment. After the collection of the macrophages, the protein levels of NLRP3 and caspase-1 in the macrophages were analyzed by Western blotting. **(E)** The expression of CaSR and NLRP3 genes in the two groups was detected (*n*=5/group). *P < 0.05, **P < 0.01, ***P < 0.001 (*t*-test).

CaSR is a calcium-sensitive receptor. To explore the role of calcium ions in the activation of NLRP3 inflammasomes, we labeled intracellular calcium with Fluo4-AM. The results show that the calcium fluorescence signal in the macrophages of the control group was 6.4, whereas that in the UPEC group was 3.75 times of that value. The fluorescence signal in the UPEC + NPS2143 group was lower than that in the infected group (**Figures 4A, B**). Compared with the control group, the UPEC group had a higher calcium level (*P* < 0.001), and the UPEC + NPS2143 group had an obviously lower calcium level (*P* < 0.05) than the UPEC group (**Figure 4C**). The expression of the NLRP3 protein in the UPEC group was visibly higher than that in the control group, and the expression of the NLRP3 protein decreased in the UPEC + NPS2143 group (**Figure 4D**). In vitro and *in vivo*, the expression levels of CaSR and NLRP3 increased obviously after UPEC stimulation (**Figures 4D, E**). In the *in vivo* and *in vitro* experiments, the highest expression levels of CaSR and NLRP3 were observed after UPEC stimulation. Thus, UPEC clearly increased the level of intracellular Ca^2+^ in the primary macrophages, and CaSR mediated the assembly of functional NLRP3 inflammasomes though Ca^2+^.

### PK2 Activates the NLRP3 Pathway Through CaSR to Increase IL-1β Secretion

**Figure 5:**
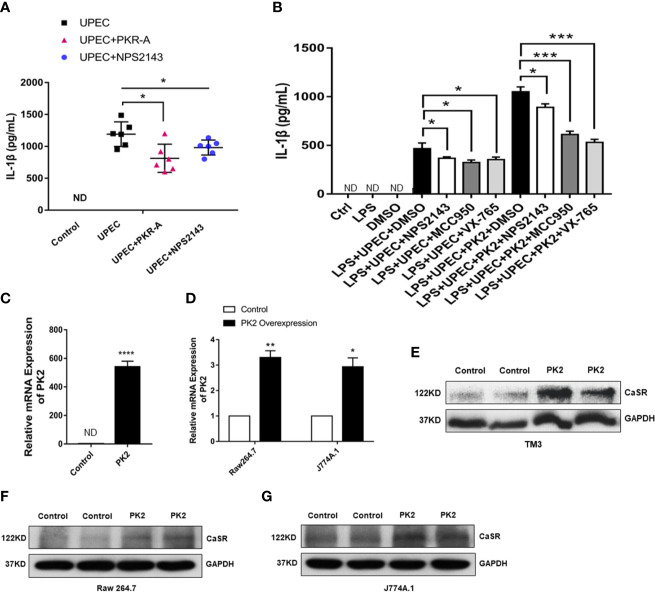
NPS2143 blocks the PK2/CaSR pathway and promotes IL-1β secretion. **(A)** Testicular interstitial fluid was collected from each group, and IL-1β level in the primary TM interstitial fluid was analyzed by ELISA (*n*= 6/group). **(B)** LPS, 5% DMSO, 20 mg/kg NPS-2143, MCC950, VX-765, LPS + UPEC + PK2 + DMSO, LPS + UPEC + PK2 + NPS2143, LPS + UPEC + PK2 + MCC950, and LPS + UPEC + PK2 + VX-765 were used to stimulate macrophages for 120 min, and cell supernatants were collected. IL-1β levels in the supernatants of the primary TM were analyzed by ELISA (*n*=5/group). MCC950 was an inhibitor of NLRP3 protein, and VX-765 was an inhibitor of Caspase-1. **(C)** Transfection efficiency of PK2 in TM3 cell line (*n*=3/group). **(D)** Transfection efficiency of PK2 in RAW 264.7 and J774A.1 (n=3/group). **(E–G)** The protein level of CaSR was detected by Western blotting. *P < 0.05, **P < 0.01, ***P < 0.001, ****P < 0.0001 (*t*-test).

PK2 and its receptors follow the G protein-coupled receptor signaling pattern, activate PK2 transcription, release functional PK2 proteins from the cytoplasm to the extracellular environment, and further activate the NLRP3 pathway. Thus, we surmised that PK2 is associated with CaSR. The ELISA results of IL-1β show that IL-1β content was not detected in the supernatant of the control group, and the secretion level of IL-1β in the cell supernatant of the UPEC group increased obviously. However, the protein level of IL-1β decreased after treatment of TM with PKRA and NPS2143. It shows that PKRA and NPS2143 exerted inhibitory effects on IL-1β secretion (**Figure 5A**). To further confirm this effect, we divided the cells into ctrl, LPS, DMSO, LPS + UPEC + DMSO, LPS + UPEC + NPS2143, LPS + UPEC + MCC950, LPS + UPEC + VX-765, and LPS + UPEC + PK2 + DMSO, LPS + UPEC + PK2 + NPS2143, and LPS + UPEC + PK2 + MCC950, LPS + UPEC + PK2 + VX-765 groups. The results demonstrate that, under the action of three inhibitors, the level of PK2 and inflammatory PK2 increased. Meanwhile, NPS2143 inhibited the secretion of the inflammatory cytokine IL-1β, and the expression level of PK2 did not change after the addition of the NPS2143 inhibitor (**Figure 5B**). We transfected the PK2 plasmid into the macrophage cell lines and TM3 cell line, and the expression level of the PK2 gene was increased approximately 560-fold in TM3 cells (**Figure 5C**) and was increased approximately 3.4-fold in TM3 cells (**Figure 5D**). Total protein extracted from the transfected cells showed that, when PK2 was overexpressed, CaSR protein expression dramatically increased (**Figures 5E–G**). In summary, PK2 is an upstream regulator of the CaSR protein, and PK2 activates the NLRP3 pathway through CaSR, thereby increasing IL-1β secretion.

## Discussion

Our results indicate that UPEC infection can induce testicular inflammation by activating the NLRP3 inflammasome in TM. Interestingly, increase in CaSR level in TM enhances this process, and calcium content in the cytoplasm of a TM infected with UPEC increases obviously. CaSR can induce the production of various proinflammatory factors. In human preadipocytes, CaSR induces TNFα, thus leading to inflammation and abnormal fat functions (19). CaSR activation can promote the secretion of the proinflammatory factor IL-6 by rat peripheral blood polymorphonuclear neutrophils (20). After CaSR activation, T lymphocyte apoptosis can be increased through the TRPC3/6-IP3 signaling pathway (21). However, CaSR can be expressed in macrophages and has corresponding functions (22). The activation of CaSR can activate the NLRP3 inflammasome in macrophages and promote the secretion of proinflammatory factors, thereby causing inflammation (23). Knocking out CaSR can reduce the activation of the NLRP3 activator by inflammatory cells (15). We detected the upregulation of CaSR mRNA and protein in the testicular tissues of the UPEC-infected rats. In addition, TM isolated from the inflammation model demonstrated increased CaSR levels. Overall, we concluded that the direct stimulation of UPEC in the testicular matrix can elevate CaSR level.

Intracellular pathways triggered by CaSR stimulation depend on cell type, and ligands and physiological conditions have been widely accepted. Previous reports on the use of CaCl_2_ to activate CaSR-dependent NLRP3 in human monocytes indicate that exposure to extracellular Ca^2+^ for 16 h triggers the proteolytic cleavage of pro-IL-1β protein (22). Data from a CaSR bias signal study indicate that Ca^2+^ alone triggers a p-ERK/ERK response, and the CaSR agonist cinacalcet can cause a higher p-ER/ERK response (24). Owing to the mobilization of Ca^2+^, CaSR activation leads to an increase in intracellular Ca^2+^ concentration. In the BMDNs of bone marrow cells, CaSR can activate NLRP3 inflammasomes by increasing the concentration of Ca^2+^ and decreasing cAMP levels and thereby regulates the secretion and maturation of IL-1β. In summary, we investigated calcium ions in the cytoplasm of rat TM induced by UPEC, and our results indicate that the calcium content in rat TM infected with UPEC obviously increased. Given the upregulation of CaSR protein in TM, we speculate that CaSR promotes the maturation and secretion of IL-1β by regulating calcium ions in the cytoplasm.

To further study their relationship, we used the CaSR inhibitor NPS2143. NPS 2143 is a novel CaSR selective antagonist with anti-inflammatory activity (25, 26). NPS 2143, on the one hand, inhibits inflammation by reducing the expression of nitric oxide synthase, cyclooxygenase 2, and NF-κB. On the other hand, NPS 2143 relieves inflammation by activating the protein kinase AMPK (27, 28). In the UPEC infection model, the testis morphology recovered, and sperm motility increased after NPS2143 treatment. In the mass spectrometry, testosterone levels were elevated in NPS2143-treated rats compared to the UPEC-infected group. In addition, the CaSR inhibitor NPS2143 decreased the intracellular calcium content. For the first time, we systematically increased the expression of CaSR mRNA and protein in the TM of UPEC-infected rats.

The innate immune response of pathogens is associated with the assembly of inflammasomes (29, 30). When NS5 binds to NLRP3, Zika-induced IL-1β release occurs in human PBMC and mouse dendritic cells (31). The bacterium Acinetobacter baumannii induces IL-1β secretion *via* the NLRP3-ASC-caspase-1 pathway, thereby causing lung injury (32). UPEC promotes the cleavage of pro-IL-1β by promoting PK2 secretion and activating the NLRP3 inflammasome (12). Despite that NLRP3 and inflammation-related IL-1β are upregulated in UPEC-infected TM, whether CaSR induces NLRP3 inflammasome activity in the pathogenesis of orchitis is unclear. The relationship between CaSR and NLRP3 inflammasomes has received considerable attention. CaSR-induced ERK1/2 signaling mediates NLRP3 activation in LS14 preadipocytes (16). In the BMDNs of bone marrow cells, CaSR activates NLRP3 inflammasomes by increasing Ca^2+^ concentration and decreasing cAMP (15). The expression of NLRP3 was identified as a key marker of NLRP3 activation (33). Thus, we speculated that CaSR can activate NLRP3 inflammatory bodies in orchitis. Consistent with our results, NLRP3 protein expression was clearly upregulated in the TM of UPEC-infected rats. NLRP3 protein levels decreased after the use of the CaSR inhibitor NPS2143. Therefore, the increase in CaSR level in the TM of the UPEC-infected rats promoted the upregulation of NLRP3 mRNA and protein, and CaSR stimulation was involved in the activation of the NLRP3 inflammasome.

PK2 activates the maturation and secretion of IL-1β through NLRP3, and PK2 along with its receptors, follow the signaling pattern of a G protein-coupled receptor. Thus, CaSR is associated with PK2. LPS and bacterial DNA directly stimulate the upregulation of PK2 in Raw 264.7 cells (34). After UPEC infection, PK2 induces the activation of the NLRP3 inflammasome through the MAPK pathway, thereby promoting the maturation of IL-1β (12). This finding is consistent with our findings. The *in vivo* and *in vitro* experiments showed that PK2 promoted the secretion and maturation of IL-1β in the immune TM. To verify the role of CaSR and PK2, we overexpressed PK2 in the TM3 cell line through the PK2 plasmid transfection method. The results demonstrate that the CaSR protein was obviously upregulated in PK2-overexpressing TM3 cells. Therefore, PK2 may activate the NLRP3 inflammasome pathway through CaSR and thereby promote IL-1β secretion. We further improved the mechanism by which testicular inflammation impairs testicular function.

**Figure 6:**
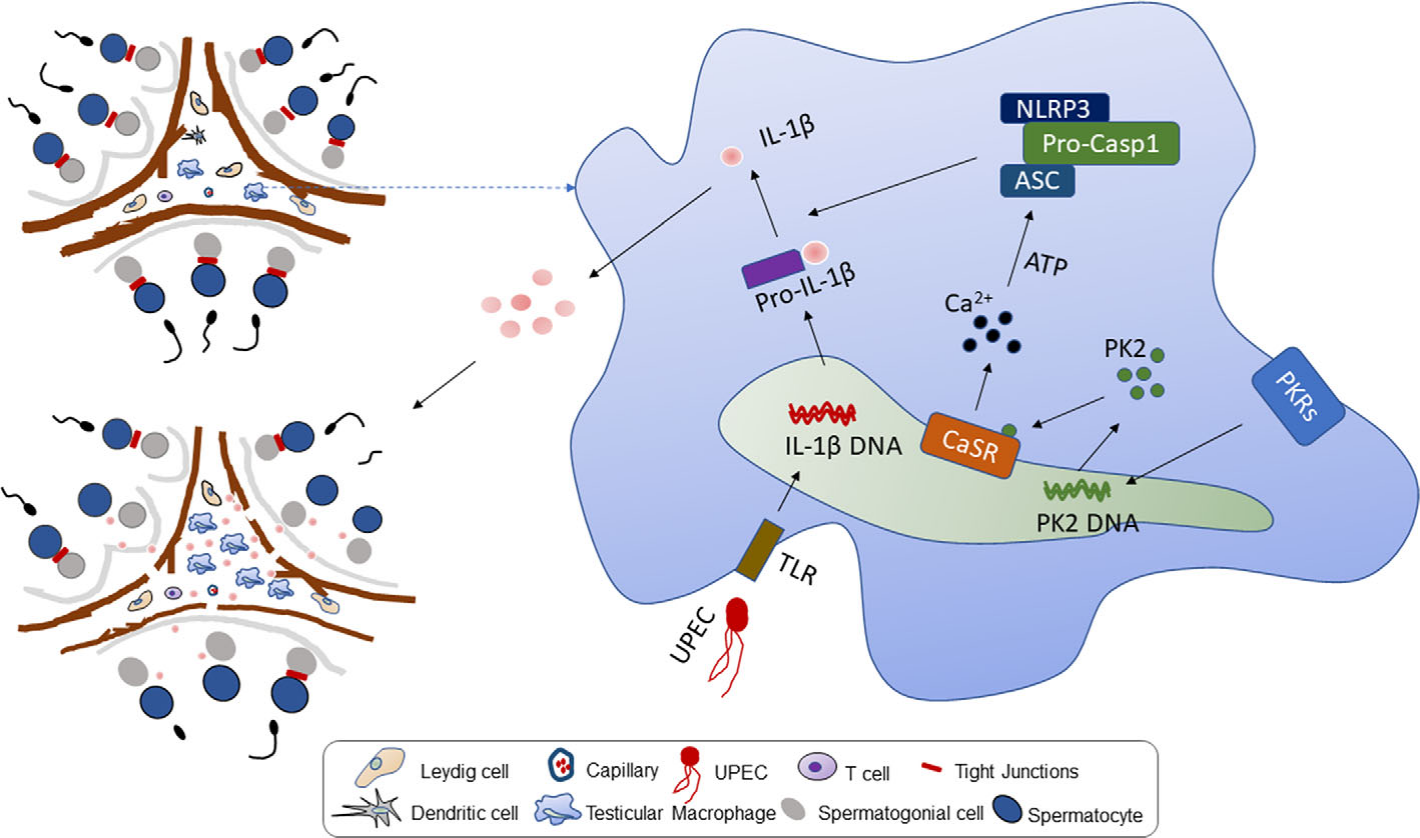
Working model of UPEC-induced Prokineticin 2 *via* CaSR activated NLRP3 inflammasome in TM. Upper left: The model diagram of rat testis structure, including two parts of spermatogenic tubule and testicular interstitium. Right: After UPEC infection, NLRP3 inflammatory bodies in rat TM are activated to secrete a large amount of proinflammatory factor IL-1β. In the process, UPEC infection induces large amounts of PK2 secreted into the cytoplasm to stimulate the activation of CaSR, and activates the NLRP3 inflammasome by increasing the level of calcium ions in the cytoplasm of macrophages. Bottom left: The large amount of proinflammatory factor IL-1β secretion leads to a decrease in testosterone levels, and the normal physiological structure of testicular tissue is destroyed.

We find that CaSR promotes the secretion of proinflammatory factor IL-1β in the NLRP3 inflammatory corpuscle pathway in orchitis and that CaSR inhibitors reduce inflammatory damage to spermatogenic epithelial cells. After NPS2143 treatment, inflammation-induced testosterone reduction was inhibited, and thus, total sperm count and forward motility increased. In the rat serum samples, the inhibitor NPS2143 increased serum testosterone levels. Overall, CaSR plays an important role in the proinflammatory process of UPEC-induced orchitis by promoting the secretion of IL-1β in TM. This study introduces the mechanism for further alleviating orchitis. After UPEC infection, PK2 induced the activation of NLRP3 inflammasomes through a CaSR mechanism and promoted IL-1β maturation. IL-1β was released from TM to the testicular stroma, affecting adjacent Leydig cells, inhibiting testosterone synthesis and leading to impaired spermatogenesis and ultimately male infertility (**Figure 6**). Our research enriches knowledge of the role of CaSR in inflammatory diseases and provides new insights into the underlying mechanisms of inflammation-related male infertility.

## Data Availability Statement

The datasets presented in this study can be found in online repositories. The names of the repository/repositories and accession number(s) can be found in the article/**Supplementary Material**.

## Ethics Statement

The animal study was reviewed and approved by the Institutional Animal Care and Use Committee of Tongji Medical College, Huazhong University of Science and Technology.

## Author Contributions

YS and KZ designed research studies, conducted experiments, analyzed data, and drafted the manuscript. YZ and ZH conducted rat model establishment, western blot, and propagation of bacteria. LH and ZF performed zebrafish assistance in immunofluorescence. WW and JX conducted sample collection and storage. CL and HZ provided intellectual input into planning of experiments and contributed to the writing of the manuscript. All authors contributed to the article and approved the submitted version.

## Funding

This work was funded by National Key R&D Program of China (2018YFC1004300, 2018YFC1004304), National Natural Foundation of China (Grant numbers: 81871148, 81701539).

## Conflict of Interest

The authors declare that the research was conducted in the absence of any commercial or financial relationships that could be construed as a potential conflict of interest.
