# Supplementary material for: Prokineticin 2 via Calcium-Sensing Receptor Activated NLRP3 Inflammasome Pathway in the Testicular Macrophages of Uropathogenic Escherichia coli-Induced Orchitis
Source: Front Immunol. 2020 Oct 23;11:570872. doi: 10.3389/fimmu.2020.570872 (PMC7644440; doi:10.3389/fimmu.2020.570872)
Supplement: Supplementary file 1 [file Presentation_1.pptx]

## Slide 1
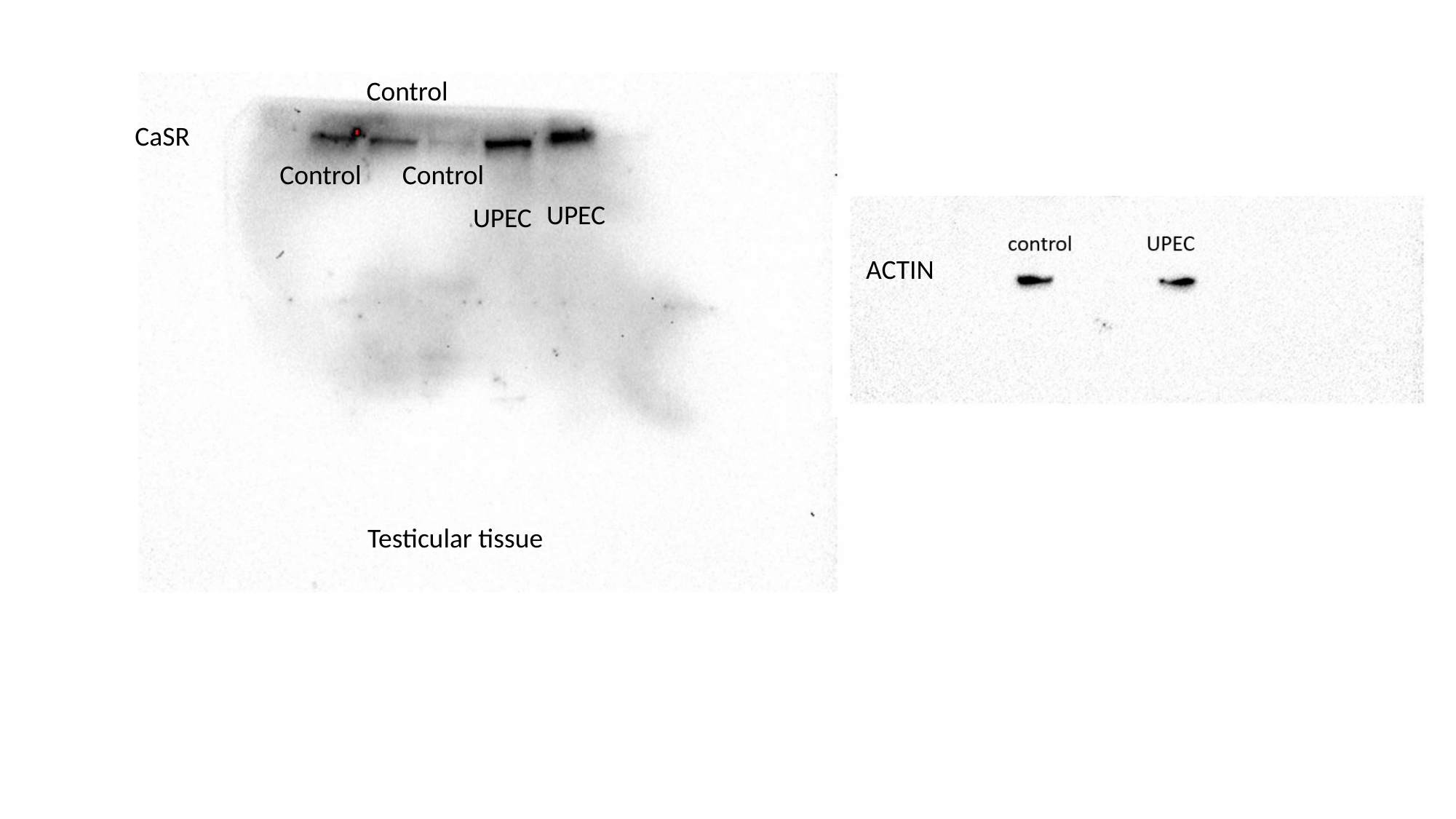

Control
CaSR
Control
Control
UPEC
UPEC
ACTIN
Testicular tissue

## Slide 2
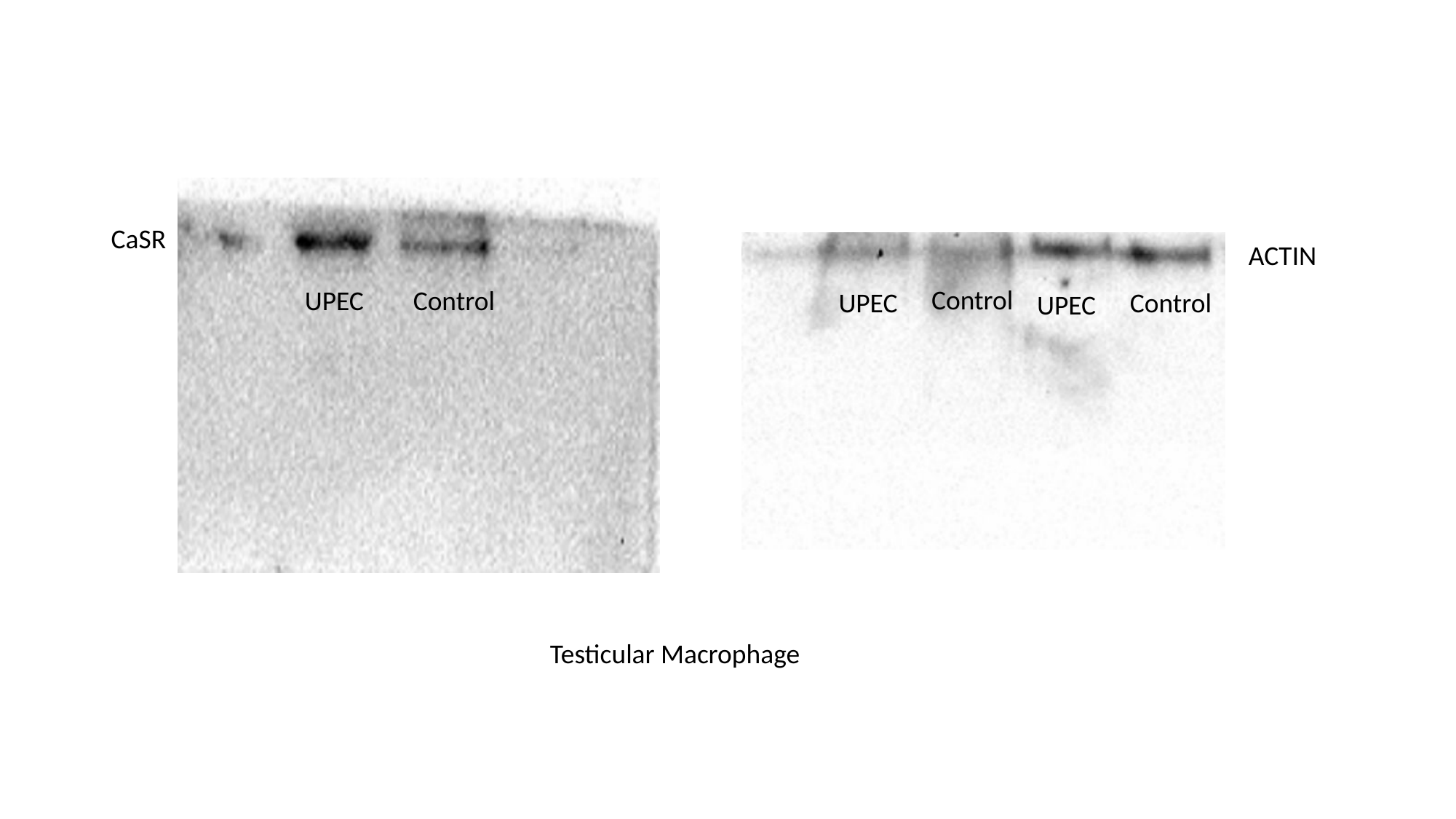

CaSR
ACTIN
Control
UPEC
Control
UPEC
Control
UPEC
Testicular Macrophage

## Slide 3
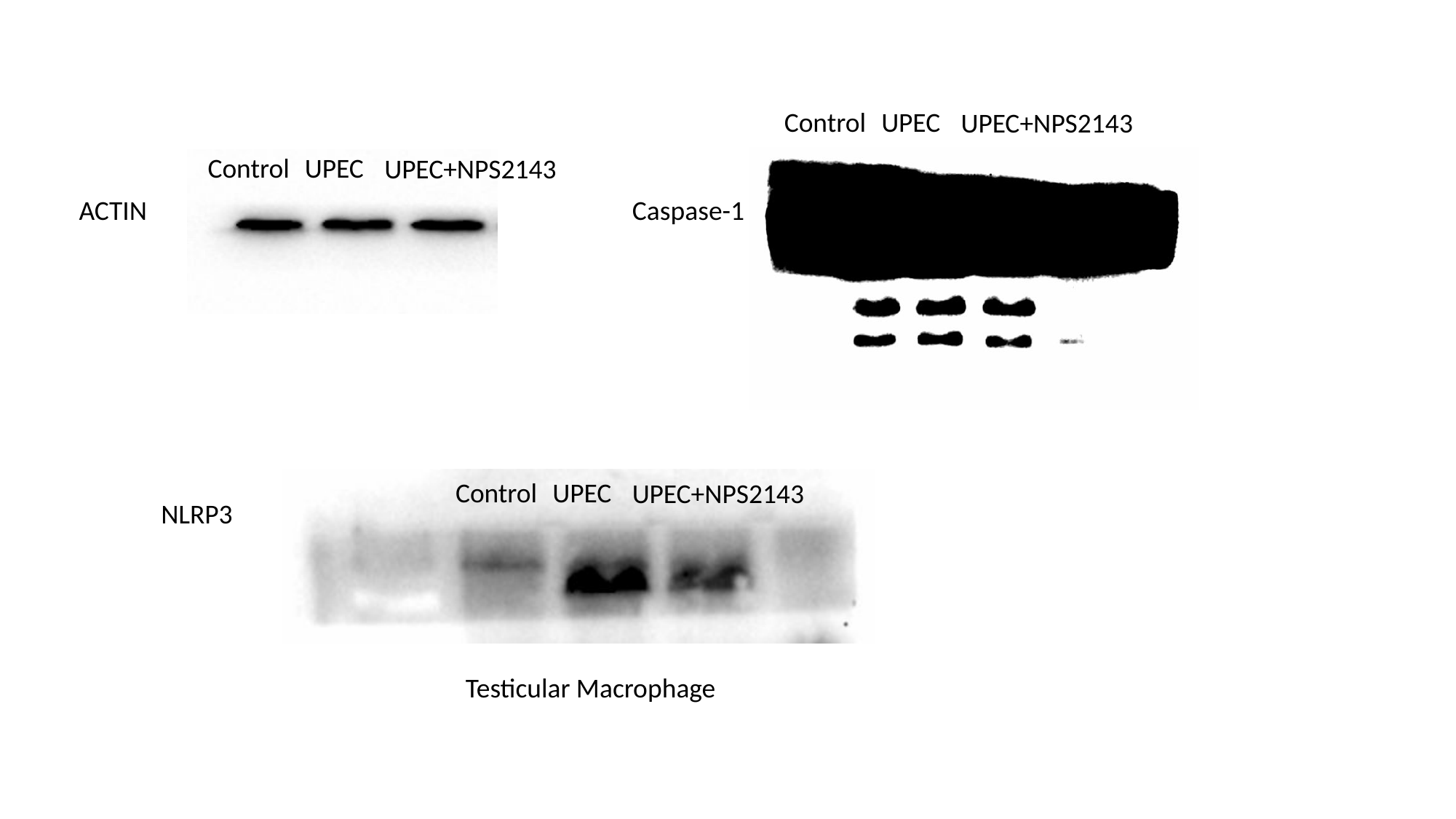

Control
UPEC
UPEC+NPS2143
Control
UPEC
UPEC+NPS2143
ACTIN
Caspase-1
Control
UPEC
UPEC+NPS2143
NLRP3
Testicular Macrophage

## Slide 4
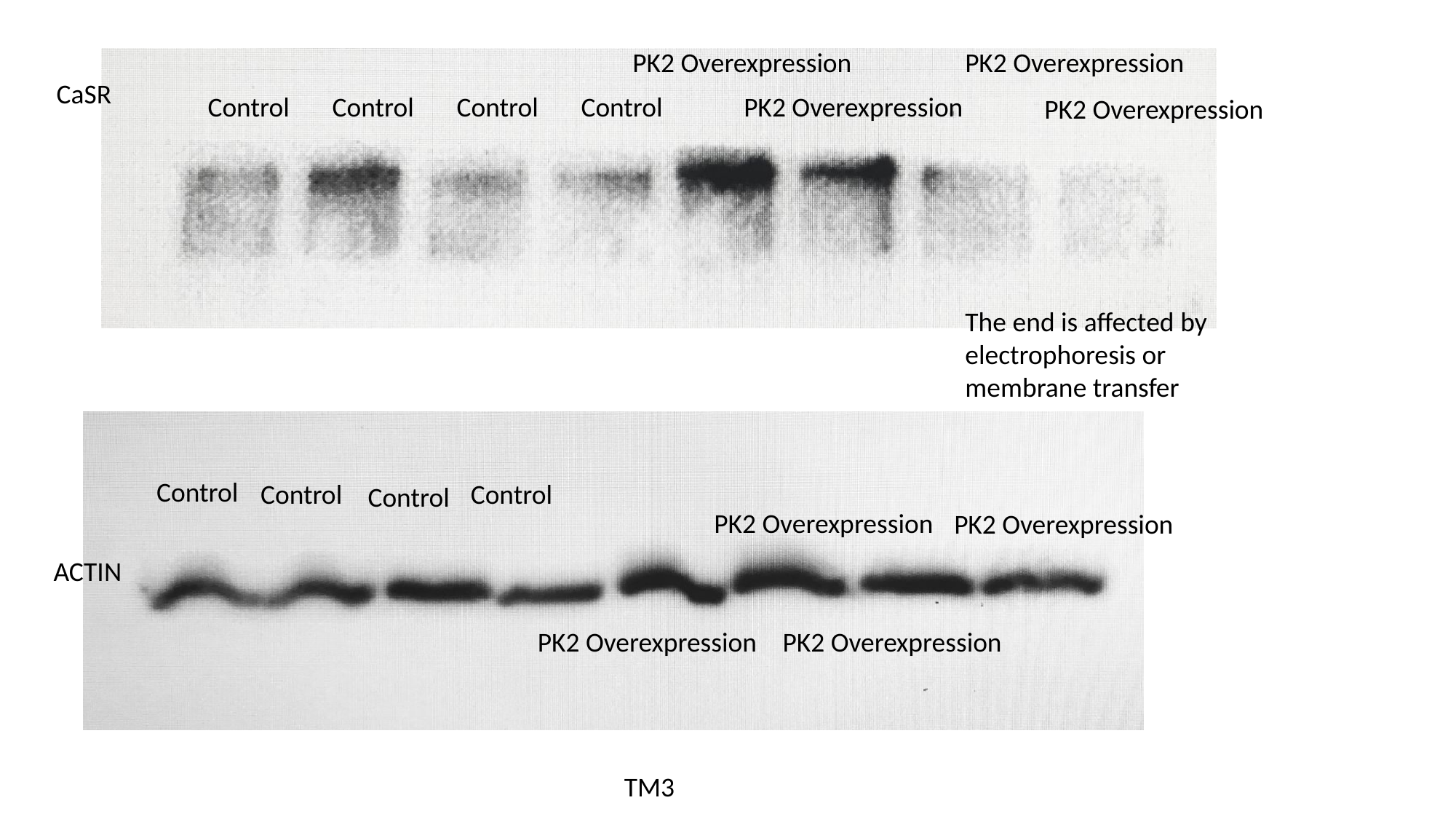

PK2 Overexpression
PK2 Overexpression
CaSR
Control
Control
Control
Control
PK2 Overexpression
PK2 Overexpression
The end is affected by electrophoresis or membrane transfer
Control
Control
Control
Control
PK2 Overexpression
PK2 Overexpression
ACTIN
PK2 Overexpression
PK2 Overexpression
TM3

## Slide 5
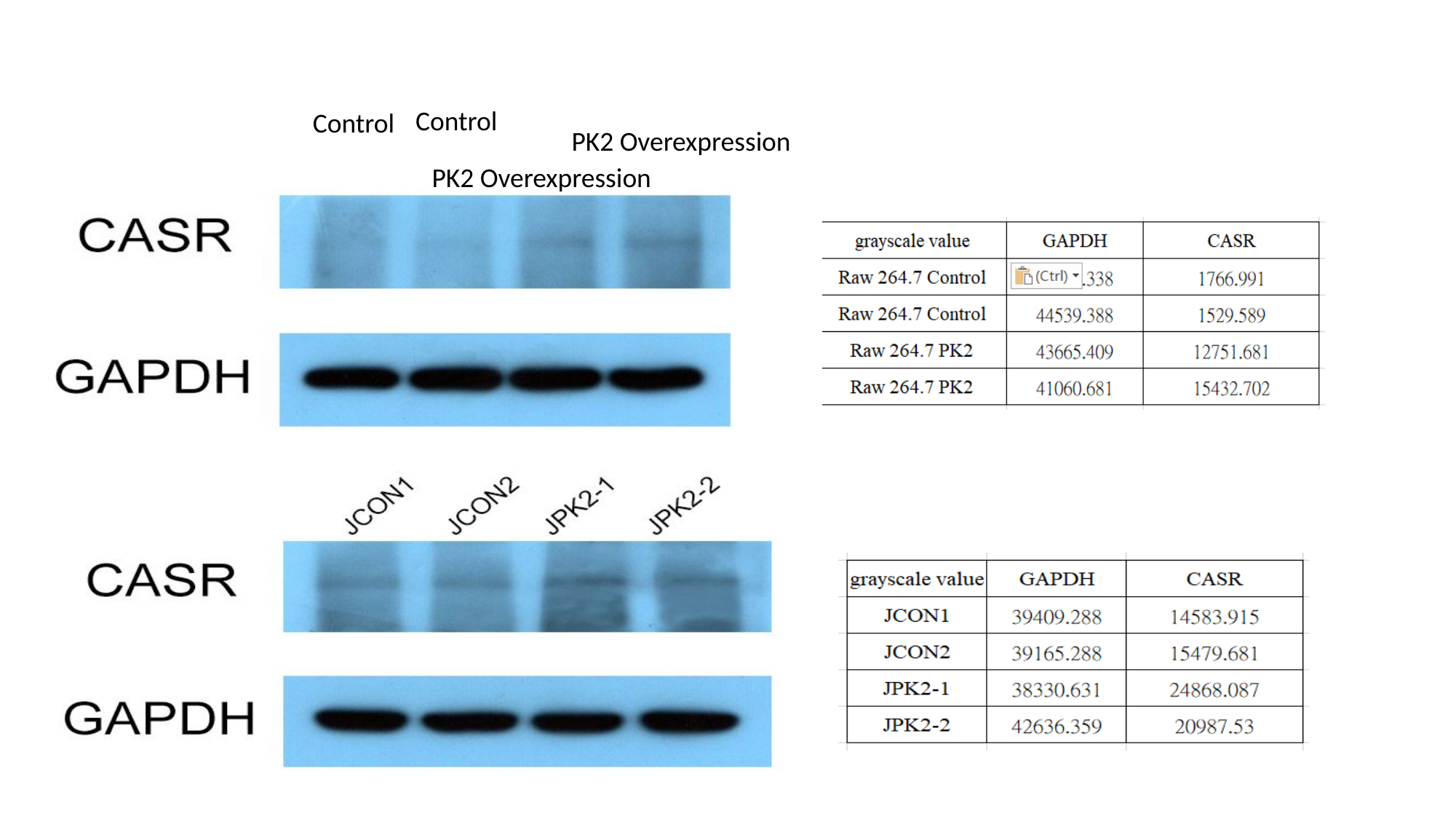

Control
Control
PK2 Overexpression
PK2 Overexpression
